# A Novel Role for Cathepsin S as a Potential Biomarker in Triple Negative Breast Cancer

**DOI:** 10.1155/2019/3980273

**Published:** 2019-06-27

**Authors:** Richard D. A. Wilkinson, Roberta E. Burden, Sara H. McDowell, Darragh G. McArt, Stephen McQuaid, Victoria Bingham, Rich Williams, Órla T. Cox, Rosemary O'Connor, Nuala McCabe, Richard D. Kennedy, Niamh E. Buckley, Christopher J. Scott

**Affiliations:** ^1^Centre for Cancer Research and Cell Biology, Queen's University Belfast, BT9 7AE, UK; ^2^School of Pharmacy, Queen's University Belfast, BT9 7BL, UK; ^3^Northern Ireland Molecular Pathology Laboratory, Queen's University Belfast, BT9 7AE, UK; ^4^School of Biochemistry and Cell Biology, University College Cork, Ireland; ^5^ALMAC Diagnostics, ALMAC Group, BT63 5QD, UK

## Abstract

Cathepsin S (CTSS) has previously been implicated in a number of cancer types, where it is associated with poor clinical features and outcome. To date, patient outcome in breast cancer has not been examined with respect to this protease. Here, we carried out immunohistochemical (IHC) staining of CTSS using a breast cancer tissue microarray in patients who received adjuvant therapy. We scored CTSS expression in the epithelial and stromal compartments and evaluated the association of CTSS expression with matched clinical outcome data. We observed differences in outcome based on CTSS expression, with stromal-derived CTSS expression correlating with a poor outcome and epithelial CTSS expression associated with an improved outcome. Further subtype characterisation revealed high epithelial CTSS expression in TNBC patients with improved outcome, which remained consistent across two independent TMA cohorts. Further* in silico* gene expression analysis, using both in-house and publicly available datasets, confirmed these observations and suggested high CTSS expression may also be beneficial to outcome in ER-/HER2+ cancer. Furthermore, high CTSS expression was associated with the BL1 Lehmann subgroup, which is characterised by defects in DNA damage repair pathways and correlates with improved outcome. Finally, analysis of matching IHC analysis reveals an increased M1 (tumour destructive) polarisation in macrophage in patients exhibiting high epithelial CTSS expression. In conclusion, our observations suggest epithelial CTSS expression may be prognostic of improved outcome in TNBC. Improved outcome observed with HER2+ at the gene expression level furthermore suggests CTSS may be prognostic of improved outcome in ER- cancers as a whole. Lastly, from the context of these patients receiving adjuvant therapy and as a result of its association with BL1 subgroup CTSS may be elevated in patients with defects in DNA damage repair pathways, indicating it may be predictive of tumour sensitivity to DNA damaging agents.

## 1. Introduction

Breast cancer is a highly heterogeneous disease and may be classified into different sub-types which affects treatment approach and patient prognosis [1]. Classification of breast cancer has been assigned via the presence/absence of the estrogen receptor (ER) or HER2 amplification, which allow use of targeted treatments such as tamoxifen and trastuzumab, respectively. Tumour cells lacking these receptors, in addition to the progesterone receptor (PR), are termed “triple negative” (TNBC) and have the poorest outcome due in part to the lack of targeted therapies available. TNBCs are therefore typically treated with a cocktail of chemotherapies such as FEC (5-*F*U,* E*pirubicin, and* C*yclophosphamide). Despite a high rate of response to chemotherapy, TNBC is associated with high rates of relapse and death [2]. This “triple negative paradox” is underpinned by a high level of molecular heterogeneity [3]. In response to this, increased efforts have been made to identify markers which may improve patient outcome following diagnosis [4, 5], not only to allow better treatment stratification but also to identify new therapeutic targets.

The cysteine protease cathepsin S (CTSS) is one of a family of 11 cysteine cathepsin proteases, and has been found to be associated with a variety of pathologies, including cancer [6, 7]. In contrast to other members of the cysteine cathepsins, CTSS is normally constrained to macrophage and lymphoid tissues. However, presence of CTSS has been observed in a number of cancer types, including prostate [8, 9], gastric [10] and hepatocellular [11] carcinomas. Furthermore, increased CTSS expression has been shown to hold prognostic value in grade IV astrocytomas [12], colorectal carcinomas [13], and gastric cancer [14], where it is associated with a poor outcome. Collectively, these observations have attracted interest in its therapeutic potential in cancer [7].

The viability of targeting this protease in cancer has been evaluated using pancreatic and colorectal carcinoma gene depletion models [15–17], and treatment with a monoclonal antibody inhibitor FSN0503 [18] and a selective small molecule inhibitor compound [19]. Inhibition/depletion of CTSS produced reductions in tumour invasion, burden, proliferation and vascularisation, as well as increased apoptosis.

Recently, Sevenich and colleagues examined the role of CTSS in breast cancer progression, identifying a role for CTSS in breast-to-brain metastases via cleavage of JAM-B, a junctional adhesion molecule involved in blood-brain barrier transmigration [17]. However, the clinical utility of CTSS as a biomarker in breast cancer has not been investigated to date. In this study we therefore aimed to understand the specific expression of CTSS, not only within epithelial and stromal compartments in breast tumours, but also the known molecular subgroups. This expression data was correlated with clinical outcome to investigate the potential prognostic and/or predictive role of CTSS.

## 2. Materials and Methods

### 2.1. Tissue Microarray Patient Sample Selection and Immunohistochemical Staining

All tissue samples were located from the Belfast and the South Eastern Health and Social Care Trust (BSHSCT) were obtained under the auspices of the Northern Ireland Biobank (NIB) (www.nibiobank.org), which has ethical approval (ref: 11/NI/0013) to collect, store and distribute de-identified/anonymised samples to researchers. The present study has ethical approval from NIB approval (reference. NIB14-0125). Tissue microarray study design, patient selection and construction of the BR300 cohort has been described previously in Boyle et al. [20]. This study was designed as outlined in Supplementary Figure 1. Briefly, the patient cohort compiled 296 female patients with* de novo *breast cancer and included matching clinical, pathological and outcome parameters. All patients within the cohort were diagnosed and received treatment in Northern Ireland, with the vast majority of the tissue resection samples obtained, processed and reported from one of the two hospitals in the Belfast catchment area between September 1997 and May 2009. All tissue data presented here was obtained by surgical resection, comprising of total or partial mastectomies with axillary node clearance. All patients present within the cohort subsequently received anthracycline-based chemotherapy with or without radiotherapy. Patients exhibiting positive hormone receptor or HER2 status were administered hormone therapy or trastuzumab. None of the patients were treated neoadjuvantly. Patient exclusion criteria included male sex and past history of any cancer type. Unique TNBC cases were collated from two independent bespoke TNBC TMA cohorts available from the NIB and previously described in Humphries et al. [21] and Orr et al. [22]. Immunohistochemical staining of CTSS was carried out in the Northern Ireland Molecular Pathology Laboratory (QUB). Sections were cut from the TMA blocks to a diameter of 4 *μ*m using a rotary microtome, dried at 37°C overnight, and then used for immunohistochemical staining with rabbit anti-human CTSS antibody (1:250) (HPA002988, Atlas Antibodies, UK) using an automated immuno-stainer (Leica Bond-Max, UK). All sections were visualised with DAB, counterstained with haematoxylin and mounted in DPX. To avoid bias, scoring was carried out by at least two independent assessors experienced in IHC analysis in breast TMAs. Preliminary analysis revealed that patients with a CTSS score of 0 and 1 behaved similarly in terms of survival, as were patients with CTSS scores of 2 and 3. Patients were therefore stratified based on low CTSS (score of 0 and 1) or high CTSS (score of 2 and 3) expression, and the effect of expression on overall survival observed.

### 2.2. Generation of Kaplan-Meier Curves for Analysis of TMA and Publicly Available Gene Datasets

Matching clinical data was obtained from the NIB upon completion of CTSS scoring. The expression data was matched with the clinical data according to the anonymous patient IDs using Microsoft Excel. Evaluation of CTSS expression on survival was completed using non-censored data, and was subsequently analysed using GraphPad Prism.

Comparative analysis of gene expression versus overall survival (2014; N=1117) and relapse free survival (N=3971) was carried out using online repository KM plotter (www.kmplot.com) [23]. Using the breast cancer dataset, survival dependent on CTSS gene expression was analysed based on intrinsic patient subtype, using a collation of previously published and publicly available Affymetrix microarray datasets, available through GEO, European Bioinformatics Institute and TCGA. Gene expression was evaluated using a median expression of CTSS probes 202901_x_at and 202902_s_at. Patient overall survival was split according to a median value cut-off point into high/low expression and all the data right-censored at 120 months (10 years). Data was obtained directly from www.kmplot.com and the figures generated using GraphPad Prism. Data was presented as percentage survival versus time in months.

### 2.3. Analysis of CTSS and Macrophage Polarisation

BR300 CTSS epithelial scores were matched with CD68, CD14 and CD163 IHC, previously stained and described by Buckley et al. [24], and split according to no CTSS expression (score = 0) or CTSS expression (score = 1-3). Analysis of macrophage polarisation by gene signatures was carried out as previously described by Jezequel et al. and Denardo et al. [25, 26], using gene expression collected and described in Buckley et al. [24].

### 2.4. Statistical Analysis

The TMA IHC clinicopathological analysis and the macrophage IHC was analysed by Chi-Square test. Differences in overall survival within the CTSS BR300 and the TNBC bespoke IHC, as well as the publically available gene expression (overall survival and relapse free survival) were evaluated by Log-Rank test and hazard ratios with 95% confidence limits reported. Statistical evaluation of CTSS expression St. Gallen and Lehmann subtypes, as well as the macrophage polarisation gene expression signatures within the BR300 cohort, were analysed using one-way ANOVA. Significance is defined as ^*∗*^p<0.05, ^*∗∗*^p<0.01, and ^*∗∗∗*^p<0.001.

## 3. Results

### 3.1. Patho-Physiological Characterisation of CTSS Expression in the Patient Samples

**Figure 1:**
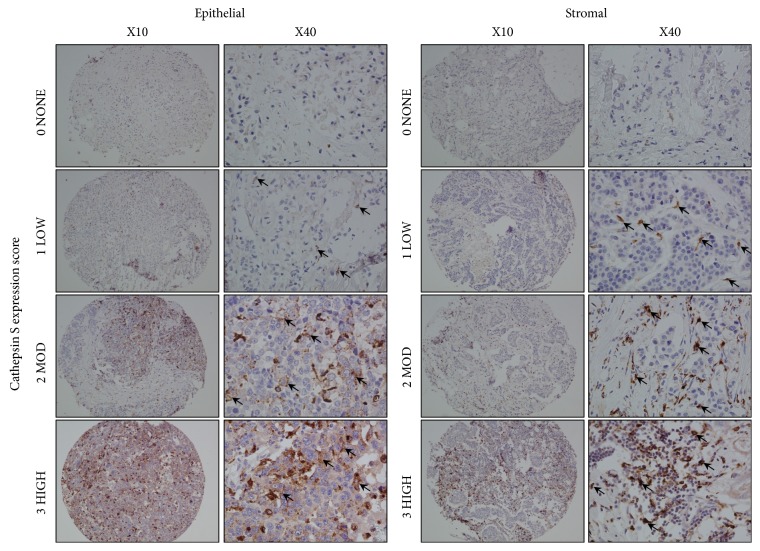
*Representative images of CTSS expression in patient samples.* CTSS-specific expression is indicated by brown staining versus blue nuclear counter staining. Samples represent either epithelial or stromal CTSS staining. Black arrows indicate areas of CTSS expression, which was separated according to high (3), moderate (2), low (1), or no expression (0).

To first investigate the role of CTSS expression in breast cancer, we applied IHC of CTSS on a tissue microarray (TMA) representing a cohort of 296 patients (hereafter referred to as BR300 cohort) [20]. Previously, several groups have indicated an importance for either tumour infiltrating lymphocyte- (TIL-) derived [27] or epithelial-derived CTSS expression [16] in tumour progression. Therefore, CTSS protein expression was evaluated for epithelial and stromal compartments separately. Based on initial assessment of staining patterns, expression was categorised as either; 0: no expression, 1: low expression, 2: moderate expression and 3: high expression, in both epithelial and stromal cells (Figure 1). When matched with the clinical data, a significant association between increased CTSS expression and tumour grade was observed in epithelial (*p=*0.0004) and stromal (*p*<0.0001) cells (Table 1). In addition, there was a significant association between high CTSS expression, and increased tumour stage (*p=*0.035) in the epithelial cells. We also observed decreased node (*p=*0.020) as well as reduced lymphovascular invasion (LVI), which approached significance, in patients with high epithelial CTSS expression. Finally, increased expression of CTSS in the stromal cells revealed a significant association with ductal breast cancer (*p*<0.0001), though it is important to note that the study is underpowered to robustly assess any association with other histologies. No significant differences were observed between histology and epithelial cell CTSS expression, and no significant difference was observed in the age of patients comparing high or low CTSS expression in either epithelial or stromal cells. Interestingly, despite no significant differences with respect to radiotherapy with CTSS expression, a significantly larger number of patients with high epithelial and stromal CTSS expression did not receive hormone therapy, suggesting a negative association between CTSS expression and ER status (*p*<0.0001) (Table 1).

### 3.2. Increased CTSS Expression in Epithelial Cells Associated with Improved Outcome

**Figure 2:**
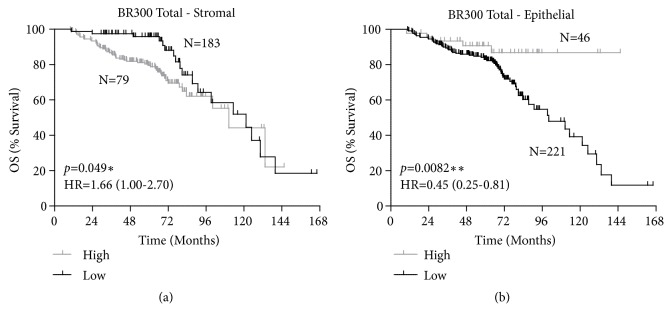
*CTSS expression is differentially associated with patient outcome based on cell compartment.* Kaplan-Meier curve stratified overall survival (OS) based on high or low CTSS expression in (a) stromal and (b) epithelial compartment. Log-Rank p-value and hazard ratio (HR) with 95% confidence intervals indicated. N=number of patients.

Preliminary analysis revealed that patients with a CTSS score of 0 and 1 behaved similarly in terms of survival, as were patients with CTSS scores of 2 and 3. Patients were therefore stratified based on low CTSS (score of 0 and 1) or high CTSS (score of 2 and 3) expression, and the effect of expression on overall survival analysed. The resulting Kaplan-Meier plots revealed distinct patterns for epithelial and stromal cell CTSS expression with respect overall survival. Consistent with previous findings, high stromal CTSS expression was associated with poor outcome (HR=1.66 (CI=1.00-2.70)* p=*0.049) (Figure 2(a)). Intriguingly, the opposite was observed with respect to high epithelial CTSS expression, which was highly significantly associated with an improved outcome (HR=0.45 (CI=0.25-0.81)* p*=0.0082) (Figure 2(b)). This led us to further investigate if the expression of epithelial-derived CTSS was specific to certain sub-types of breast cancer.

### 3.3. Increased Epithelial Cell CTSS Expression Is Associated with Improved Outcome in Triple Negative Breast Cancer

Following evaluation of CTSS protein expression and the association with survival using the BR300 patient cohort, we next wished to observe differential CTSS expression within breast cancer subtypes. Patients were subdivided into their respective subtypes according to St. Gallen classification [28]. While high CTSS was associated with good outcome, there were very few cases, which prohibited further robust analysis (Supplementary Figures 2(a)-2(e)).

**Figure 3:**
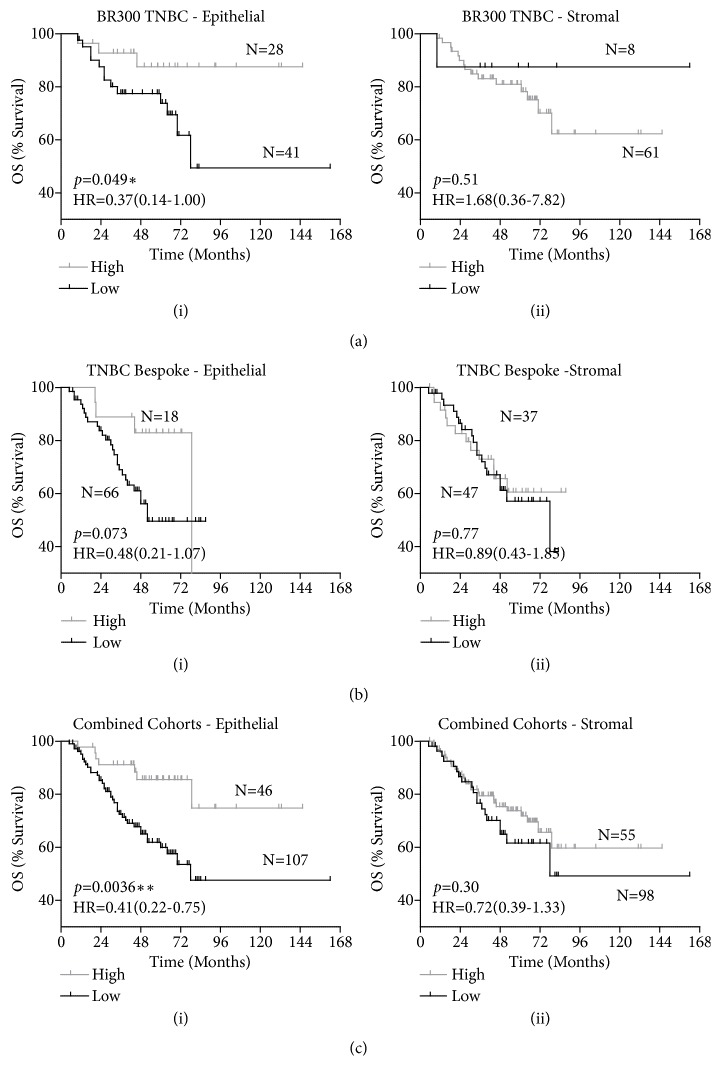
*Increased epithelial cell CTSS expression is associated with improved outcome in TNBC.* Kaplan-Meier curves stratifying overall survival (OS) of (a) the BR300 TNBC patients-alone, (b) the bespoke TNBC enriched cohort-alone, and (c) the combined BR300 and bespoke TNBC cohorts, based on high or low CTSS expression in the (i) epithelial and (ii) stromal compartment. Log-Rank p-value and hazard ratio (HR) with 95% confidence intervals indicated. N=number of patients.

Interestingly, matching outcome data for the triple negative breast cancer patients (N=69) to the CTSS epithelial expression revealed an association of high CTSS expression with a significantly improved outcome (HR=0.37 (CI=0.14-1.00)* p=*0.049) (Figure 3(a)(i)). Analysis of stromal CTSS expression revealed a non-significant trend towards poor outcome which may be due to the low number of patients within the low CTSS expression arm (HR=1.68 (CI=0.36-7.82)* p*=0.51) (Figure 3(a)(ii)).

To supplement this observation, scoring of CTSS in a bespoke triple negative breast cancer cohort (N=84) was carried out [21, 22]. Analysis of the stromal CTSS expression revealed no significant difference to outcome, but reassuringly, a trend complementing the outcome in the BR300 cohort was observed, with high CTSS epithelial expression demonstrating improved outcome in triple negative breast cancer patients, however, given the relatively small size of this cohort, significance was not quite reached (*p=*0.073) (Figure 3(b)). To enhance statistical power, the two cohorts were combined and as a result demonstrated a clear and significant improvement for TNBC patients with epithelial derived CTSS expression (HR=0.41 (CI=0.22-0.75)* p=*0.0036) (Figure 3(c)(i)) in contrast to stromal CTSS expression which showed no significant difference to outcome (Figure 3(c)(ii)).

### 3.4. Increased CTSS Gene Expression Associated with Improved Outcome in Triple Negative Breast Cancer

**Figure 4:**
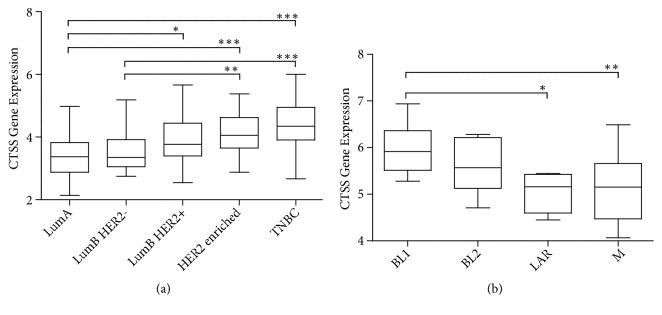
*CTSS gene expression is highest in BR300 TNBC subtype and associated with DNA damage/cell cycle pathways.* CTSS gene expression was evaluated using an in house dataset containing 300 breast cancer patients. Analysis revealed (a) CTSS expression to be highest in TNBC. (b) Lehman subgroups analysis of the TNBC patients revealed an association with the BL1 group which encompasses DNA damage and cell cycle pathways. Significance for both panels was determined by one-way ANOVA. ^*∗*^p<0.05, ^*∗∗*^p<0.01, and ^*∗∗∗*^p<0.001.

Given the results from the TMA analysis, we investigated if CTSS gene expression could also predict outcome. This allowed us to interrogate the role of CTSS further using publicly available gene expression datasets. We first validated the TMA findings using a gene expression dataset matched to the BR300 cohort [29]. Consistent with the IHC analysis, we observed CTSS to be expressed highest in TNBC (Figure 4(a)). Lehman subtype analysis of the TNBC subgroup revealed CTSS expression varied significantly across all subgroups with the highest expression observed in the IM group and lowest expression in the LAR and M groups (Figure 4(b) and Supplementary Figure 3). Refinement of the Lehmann subtype study has since shown that the molecular signatures defining the IM and MSL groups were derived from infiltrating lymphocyte and stromal cells [30]. Therefore the high CTSS expression in the IM group is most likely associated with tumour associated immune cells. Analysis of CTSS across the four epithelial-derived subgroups displayed a significant variation in expression as a whole with expression in the BL1 subtype significantly higher than the LAR and M subtypes. Of note, the BL1 subgroup is also associated with improved outcome [30].

**Figure 5:**
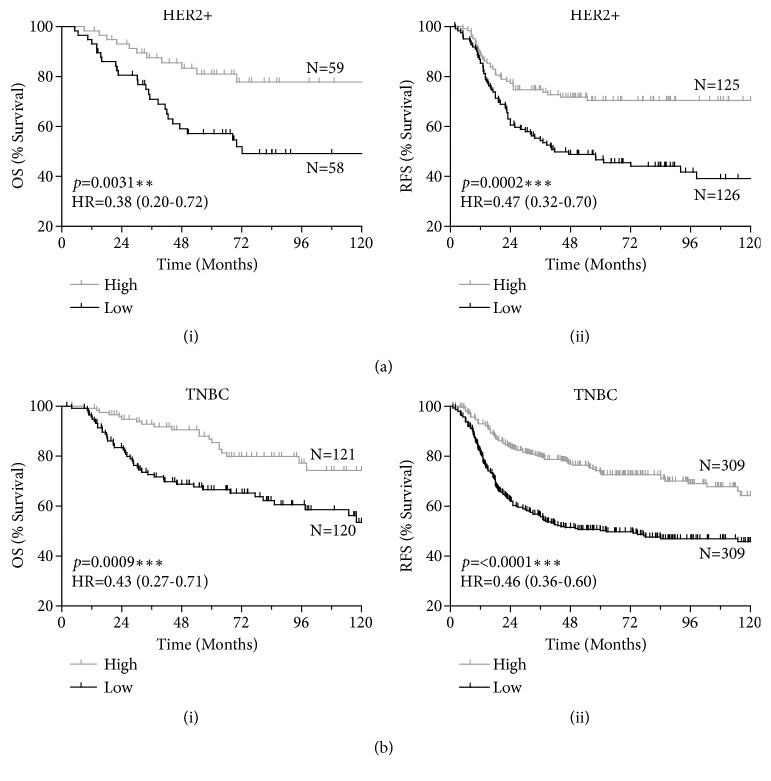
*Analysis of publicly available gene expression datasets reveal improved outcome with high CTSS expression in HER2+ and TNBC patients.* Kaplan-Meier curves stratifying (a) HER2+ and (b) TNBC patients based on high or low CTSS expression and evaluating (i) overall survival (OS) and (ii) relapse free survival (RFS). Log-Rank p-value and hazard ratio (HR) with 95% confidence intervals indicated. N=number of patients.

To supplement these observations made with our in-house patient dataset, evaluation of the relationship of gene expression and survival was carried out with publicly available datasets using KM Plotter [23]. The results indicated no significant differences between high and low* CTSS* gene expression on overall survival (OS) or relapse free survival (RFS) of breast cancer patients as a whole (Supplementary Table 1; Supplementary Figure 4). Further dissection of* CTSS* expression with respect to individual subtypes revealed no significant difference between high and low* CTSS* expression on either OS or RFS in luminal A, nor luminal B (Supplementary Table 1; Supplementary Figure 4). However, a striking significant correlation between CTSS and survival was observed in HER2+ (OS HR=0.38 (CI=0.20-0.71)* p=*0.0031, RFS HR=0.47 (0.32-0.70)* p*=0.0002) and TNBC patients (OS HR=0.43 (CI=0.27-0.71)* p=*0.0009, and RFS HR=0.46 (0.36-0.60)* p*<0.0001) (Figure 5) (Supplementary Table 1).

### 3.5. Expression of CTSS in TNBC Epithelial Cells Is Associated with the Enhanced Presence of M1 Macrophages

In order to understand some of the molecular pathology underpinning the observed association between CTSS and good outcome in TNBC patients, we interrogated the tumour microenvironment for possible clues. Given the association between CTSS expression with macrophages, we decided to examine the expression of activated M1 (tumour destructive) or alternatively activated M2 (tumour protective) macrophage polarisation markers in the context of CTSS expression.

**Figure 6:**
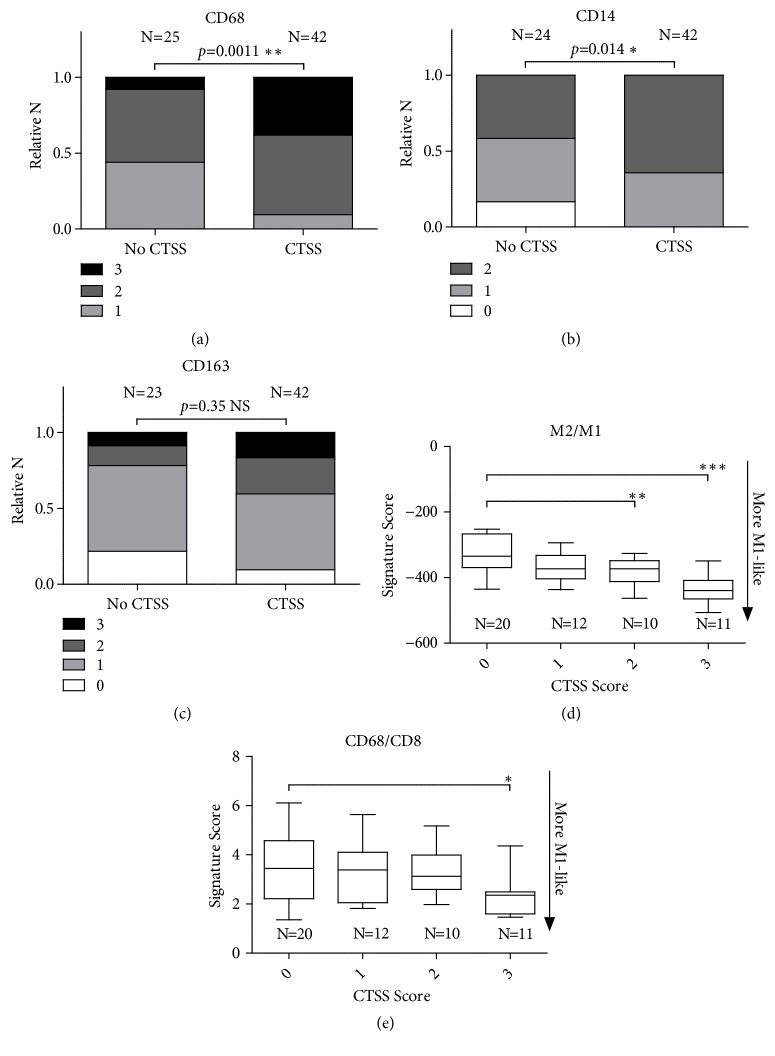
*Epithelial cell CTSS expression in TNBC patients is associated with an M1 macrophage phenotype.* Immunohistochemical epithelial CTSS scores were matched with (a) macrophage marker CD68, (b) M1 polarisation marker CD14 and (c) M2 polarisation marker CD163. Shading indicates proportion of IHC score for each marker. Statistical significance determined by Chi-Square analysis. Macrophage polarisation was analysed using gene expression algorithms and correlated with CTSS IHC expression generating (d) M2/M1 and (e) CD68/CD8 signature scores. Statistical significance determined by one-way ANOVA. N=number of patients. ^*∗*^p<0.05, ^*∗∗*^p<0.01, and ^*∗∗∗*^p<0.001.

IHC scoring of CTSS in the TNBC epithelial cells correlated significantly with an increased expression of macrophage marker CD68 (*p=*0.0011), indicative of increased macrophage infiltration (Figure 6(a)). With this increased presence of macrophages, there was a significant increase in M1 marker CD14 (*p=*0.014) (Figure 6(b)), and no significant change in the expression of M2 marker CD163 (Figure 6(c)). Using two gene expression based algorithms [25, 26], previously utilised in our TNBC cohort [24], analysis revealed a significant enhancement in M1-like phenotype with presence of epithelial CTSS expression (*p*<0.001 and* p*<0.05) (Figures 6(d) and 6(e)).

Taken together, this suggests increased expression of CTSS in the epithelial cells associates with increased infiltration of M1 polarised macrophages thus resulting a more immunocompetent microenvironment, and rationalises the improved survival observed with epithelial CTSS expression in the TNBC sub-type.

## 4. Discussion

In this study we have demonstrated a multifaceted role for CTSS as a biomarker in TNBC. This investigation began by observing differences in patient outcome based on CTSS expression, with stromal-associated CTSS expression shown to be associated with a poor outcome, whereas high CTSS expression in epithelial cells is associated with an improved outcome. Interestingly, the observation of epithelial CTSS expression in TNBC patients revealed an association with improved outcome, which remained consistent in gene expression analysis. We furthermore observed increased M1 polarisation of macrophage in patients exhibiting high CTSS expression in the epithelial cells. Taken together, we found differential CTSS expression had compartmental and sub-type effects on patient outcome, highlighting a potentially novel role for this protease in TNBC.

Due to their potent and promiscuous proteolytic function, cysteine cathepsins have previously been implicated in a number of pathological roles as a result of extracellular matrix remodelling including angiogenesis, invasion and metastases [7]. As such, increased CTSS expression has been shown be associated with poor clinical features in a number of cancer types [8–11], as well as holding prognostic value with expression associated with poor outcome in others [12, 13]. To the best of our knowledge, this is the first investigation of CTSS expression in breast cancer using clinical samples evaluating clinical outcome, and furthermore, accounting for outcome based on epithelial or stromal CTSS expression.

We observed clinicopathological patterns with respect CTSS expression and compartment type. When comparing high versus low CTSS scores, we observed an increase in the number of patients with grade 3 tumours with high CTSS expression in both epithelial (53.39% low versus 84.78% high) and stromal (39.24% low versus 67.76% in high) cells. Furthermore, patients with high epithelial CTSS expression also demonstrated decreased node scoring and LVI positivity, indicators of improved outcome. In the epithelial cells, a significant association with increased tumour stage was also observed with high CTSS expression. These are consistent with observations made elsewhere regarding CTSS expression in breast cancer [31]. Interestingly, high CTSS expression in infiltrating cells was associated with poor outcome. This is consistent with the increased aberrant expression of this protease in other carcinomas where as a result of pro-tumorigenic role of this protease in cancer, increased CTSS has been associated with poor outcome reported as a result of tumour associated macrophages (TAMs) [15]. In contrast to this, CTSS expression in the epithelial cells displayed an opposite phenotype. Upon further analysis of subtypes, and within the context of this cohort, we found high CTSS expression to be associated with improved outcome with epithelial CTSS expression in TNBC patients. Taken altogether, this indicates a dual role for this protease in tumour development based on compartmental and subtype expression.

Further* in silico* analysis confirmed these clinical outcome findings, and also highlighted a potential association of CTSS and outcome in the HER2+ subgroup, suggesting a role for CTSS as a biomarker in ER- disease as a whole. This complements the increased number of patients with high epithelial CTSS who did not receive adjuvant hormone therapy (78%), suggesting these tumours exhibit an ER- background. Interestingly, Gautam and colleagues also observed an association between high epithelial CTSS expression and ER- subtype [31]. From a clinical perspective, patients with ER- breast cancers are significantly associated with poor outcome compared to ER+ breast cancer patients, as they lack the relevant targets for therapy [32–36]. Consequentially, there is a real need to stratify these patients further, to maximise improved outcome in patients. Based on our observations, there may be value in further characterising CTSS expression in a larger ER- patient comparative cohort, as this protease may hold utility as a prognostic biomarker in this setting.

An important feature of this study was the use of patient samples who all received adjuvant treatment prior to collection of tumour resections. Analysis of our in-house TNBC gene expression dataset using the Lehmann subgroups demonstrated an association of CTSS expression with the BL1 group, characterised by defects in DNA damage repair pathways. Interestingly, previous investigation using publicly available gene datasets representing 300 TNBC patients who received neo-adjuvant chemotherapeutic treatment, revealed the BL1 subgroup to display the greatest pathological complete response versus the BL2 and LAR patient subgroups [30]. This suggests that expression of CTSS in epithelial cells may be associated with tumours defective in DNA damage repair, and therefore, indicates CTSS expression may be predictive of sensitivity to DNA damaging chemotherapies. Whilst this study has focused on IHC analysis of resected tumours, others have demonstrated that CTSS levels can be detected in patient serum for a variety of diseases [7]. Considering the suggested link between CTSS expression from a prognostic and predictive perspective, it may be of interest to further investigate in liquid biopsies.

The relevance of the tumour immune microenvironment is becoming more important with the development of therapeutic strategies to target this compartment [37]. Consequentially, appreciation of underlying biological associations between tumour and immune cells may help better guide therapies in the future. Here we show a positive correlation between TNBC epithelial derived CTSS expression and a more favourable M1 microenvironment. The relationship between CTSS and TAMs has been widely reported using* in vivo* models [16, 17, 27, 38–40]. These studies have highlighted the relevance of macrophages as a source of CTSS at the tumour site. Furthermore, these studies have associated an M2-marcophage phenotype (tumour protective), and have implicated CTSS in a modulating role via an autophagy-mediated mechanism [40, 41]. Interestingly, the epithelial CTSS expression in TNBC patients demonstrated an enriched M1 polarisation phenotype, consistent with the observed improved outcome. We believe this underlines a more complex relationship between tumour epithelial and stromal cell compartments than has been demonstrated in pre-clinical models, and possibly between cancer types, highlighting a need for further in-depth analysis in patient samples.

## 5. Conclusion

In conclusion, we have characterised the expression profile of CTSS in breast cancer patient samples and have found that both compartmental and subtype expression of this protease can affect patient outcome. This study highlights a need for further investigation into this protease within breast cancer, to consolidate the potential predictive and prognostic utility of CTSS expression in different subtypes. Furthermore, a deeper appreciation of the biology underlying this disease will help guide treatment regimens and possible application of CTSS inhibitors in the future.

## Figures and Tables

**Table 1:** *Clinicopathological information for BR300 tissue microarray categorised according to compartmental CTSS scores.* CTSS scores of 0 and 1 behaved similarly in terms of survival, as were patients with CTSS scores of 2 and 3. Patients were therefore stratified based on low CTSS (score of 0 and 1) or high CTSS (score of 2 and 3) expression. Differences between clinical information was evaluated based on high and low CTSS scores in either the epithelial and stromal compartments. Statistical significance determined by Chi-Square test. Figure in brackets indicates percentage of total. LVI=lymphovascular invasion. N=number of patients.

BR300 Cohort		CTSS Epithelial				CTSS Stromal			
		N (%)	Low (%)	High (%)	*p*-value	N (%)	Low (%)	High (%)	*p*-value
Characteristic		267 (100)	221 (100)	46 (100)		262 (100)	79 (100)	183 (100)	

Age	N≤51	140 (52)	113 (51)	27 (59)		140 (53)	41 (52)	99 (54)	
Median = 51	N>51	127 (48)	108 (49)	19 (41)	0.42	122 (47)	38 (48)	84 (46)	0.79

Grade	1	4 (1)	4 (2)	0 (0)		4 (2)	3 (4)	1 (1)	
	2	106 (40)	99 (45)	7 (15)		103 (39)	45 (57)	58 (32)	
	3	157 (59)	118 (53)	39 (85)	0.0004^*∗∗∗*^	155 (59)	31 (39)	124 (68)	<0.0001^*∗∗∗*^

Tumour	1	54 (20)	45 (20)	9 (20)		54 (21)	14 (18)	40 (22)	
	2	171 (64)	146 (66)	25 (54)		166 (63)	52 (66)	114 (62)	
	3	36 (13)	24 (11)	12 (26)		36 (14)	11 (14)	25 (14)	
	4/4b	6 (2)	6 (3)	0 (0)	0.035^*∗*^	6 (2)	2 (3)	4 (2)	0.9

Node	0	114 (43)	86 (39)	28 (61)		113 (43)	33 (42)	80 (44)	
	1	93 (35)	83 (38)	14 (30)		91 (35)	26 (33)	64 (35)	
	2	34 (13)	31 (14)	3 (7)		33 (13)	13 (16)	20 (11)	
	3	26 (10)	25 (11)	1 (2)	0.020^*∗*^	26 (10)	7 (9)	19 (10)	0.66

LVI	Yes	168 (63)	145 (66)	23 (50)		164 (63)	51 (65)	113 (62)	
	No	96 (36)	73 (33)	23 (50)		96 (37)	26 (33)	70 (38)	
	Unknown	3 (1)	3 (1)	0 (0)	0.076	3 (1)	2 (3)	1 (1)	0.3

Histology	Ductal	210 (79)	172 (78)	38 (83)		207 (79)	49 (62)	158 (86)	
	Lobular	27 (10)	24 (11)	3 (7)		26 (10)	16 (20)	10 (5)	
	Mixed	24 (9)	21 (10)	3 (7)		23 (9)	12 (15)	11 (6)	
	Other	6 (2)	4 (2)	2 (4)	0.52	6 (2)	2 (3)	4 (2)	<0.0001^*∗∗∗*^

Radiotherapy	Yes	220 (82)	182 (82)	38 (83)		215 (82)	70 (89)	145 (79)	
	No	47 (18)	39 (18)	8 (17)	1.00	47 (18)	9 (11)	38 (21)	0.080

Hormone	Yes	157 (59)	147 (67)	10 (22)		157 (60)	65 (82)	88 (48)	
Therapy	No	110 (41)	74 (33)	36 (78)	<0.0001^*∗∗∗*^	109 (42)	14 (18)	95 (52)	<0.0001^*∗∗∗*^

## Data Availability

All TMA samples are available upon application from the Northern Ireland Biobank (http://www.nibiobank.org).

## Abbreviations

BL1: Basal-like 1
BL2: Basal-like 2
BR300: Breast 300 cohort
CI: 95% Confidence interval
CTSS: Cathepsin S
ER: Estrogen receptor
HR: Hazard ratio
IHC: Immunohistochemistry
IM: Immunomodulatory
LAR: Luminal Androgen Receptor
LVI: Lymphovascular invasion
M: Mesenchymal
MSL: Mesenchymal stem cell-like
N: Number of patients
NIB: Northern Ireland Biobank
OS: Overall survival
RFS: Relapse free survival
TAM: Tumour associated macrophage
TMA: Tissue microarray
TNBC: Triple negative breast cancer.

## Additional Points

The article by Lewis and colleagues should be cited in any published manuscripts which may arise from the use of these samples [42].

## Supplementary Materials

Supplementary Materials
* Supplementary Figure 1: *Flowchart describing study design.* Supplementary Figure 2:* Breakdown of CTSS scores across subtypes and associated Kaplan-Meier OS figures. (a) Percentage breakdown of patients with high and low CTSS in (i) epithelial and (ii) stromal compartments. Kaplan-Meier curve of (b) Luminal A, (c) Luminal B/HER2-, (d) Luminal B/HER2+, and (e) HER2+ breast cancer subtypes overall survival (OS) stratified based on high or low CTSS expression in (i) stromal and (ii) epithelial compartment. Log-Rank p-value indicated. N=number of patients.* Supplementary Figure 3:* Complete Lehman analysis figure of subtype CTSS gene expression.* Supplementary Figure 4:* No effect on OS or RFS in total, Luminal A, or Luminal B breast cancer subtypes stratified by gene expression. Kaplan-Meier curves stratifying (a) total breast cancer patient population, (b) luminal A patient population, and (c) luminal B patient population, based on high or low CTSS gene expression evaluating (i) overall survival (OS) and (ii) relapse free survival (RFS). Log-rank p-value and hazard ratio (HR) indicated. N=number of patients.* Supplementary Table 1:* Increased CTSS gene expression associated with improved survival in HER2+ and Triple Negative breast cancers. Analysis of publicly available gene expression data with matching clinical outcome from KM plotter revealed a significant association of increased CTSS expression with improved overall survival and relapse free survival. N=number of patients. HR= hazard ratio. CI=95% confidence intervals.

## References

[1] Dai X., Li T., Bai Z. (2015). Breast cancer intrinsic subtype classification, clinical use and future trends. *American Journal of Cancer Research*.

[2] Liedtke C., Mazouni C., Hess K. R. (2008). Response to neoadjuvant therapy and long-term survival in patients with triple-negative breast cancer. *Journal of Clinical Oncology*.

[3] Carey L. A., Dees E. C., Sawyer L. (2007). The triple negative paradox: primary tumor chemosensitivity of breast cancer subtypes. *Clinical Cancer Research*.

[4] Yadav B. S., Chanana P., Jhamb S. (2015). Biomarkers in triple negative breast cancer: a review. *World Journal of Clinical Oncology*.

[5] Cox O. T., Edmunds S. J., Simon-Keller K. (2019). PDLIM2 is a marker of adhesion and *β*-catenin activity in triple-negative breast cancer. *Cancer Research*.

[6] Small D. M., Burden R. E., Scott C. J. (2011). The emerging relevance of the cysteine protease cathepsin S in disease. *Clinical Reviews in Bone and Mineral Metabolism*.

[7] Wilkinson R. D., Williams R., Scott C. J., Burden R. E. (2015). Cathepsin S: therapeutic, diagnostic, and prognostic potential. *Biological Chemistry*.

[8] Lindahl C., Simonsson M., Bergh A. (2009). Increased levels of macrophage-secreted cathepsin S during prostate cancer progression in TRAMP mice and patients. *Cancer Genomics & Proteomics*.

[9] Fernández P. L., Farré X., Nadal A. (2001). Expression of Cathepsins B and S in the progression of prostate carcinoma. *International Journal of Cancer*.

[10] Yang Y., Kiat L. S., Yee C. L. (2010). Cathepsin S mediates gastric cancer cell migration and invasion via a putative network of metastasis-associated proteins. *Journal of Proteome Research*.

[11] Xu J., Li D., Ke Z., Liu R., Maubach G., Zhuo L. (2009). Cathepsin S is aberrantly overexpressed in human hepatocellular carcinoma. *Molecular Medicine Reports*.

[12] Flannery T., McQuaid S., McGoohan C. (2006). Cathepsin S expression: An independent prognostic factor in glioblastoma tumours - A pilot study. *International Journal of Cancer*.

[13] Gormley J. A., Hegarty S. M., O’Grady A. (2011). The role of Cathepsin S as a marker of prognosis and predictor of chemotherapy benefit in adjuvant CRC: a pilot study. *British Journal of Cancer*.

[14] Liu W., Liu D., Cheng K. (2016). Evaluating the diagnostic and prognostic value of circulating cathepsin S in gastric cancer. *Oncotarget*.

[15] Gocheva V., Zeng W., Ke D. (2006). Distinct roles for cysteine cathepsin genes in multistage tumorigenesis. *Genes & Development*.

[16] Small D. M., Burden R. E., Jaworski J. (2013). Cathepsin S from both tumor and tumor-associated cells promote cancer growth and neovascularization. *International Journal of Cancer*.

[17] Sevenich L., Bowman R. L., Mason S. D. (2014). Analysis of tumour- and stroma-supplied proteolytic networks reveals a brain-metastasis-promoting role for cathepsin S. *Nature Cell Biology*.

[18] Burden R. E., Gormley J. A., Jaquin T. J. (2009). Antibody-mediated inhibition of cathepsin S blocks colorectal tumor invasion and angiogenesis. *Clinical Cancer Research*.

[19] Wilkinson R. D., Young A., Burden R. E., Williams R., Scott C. J. (2016). A bioavailable cathepsin S nitrile inhibitor abrogates tumor development. *Molecular Cancer*.

[20] Boyle D. P., McArt D. G., Irwin G. (2014). The prognostic significance of the aberrant extremes of p53 immunophenotypes in breast cancer. *Histopathology*.

[21] Humphries M. P., Hynes S., Bingham V. (2018). Automated tumour recognition and digital pathology scoring unravels new role for PD-L1 in predicting good outcome in ER-/HER2+ breast cancer. *Journal of Oncology*.

[22] Orr K., Buckley N. E., Haddock P. (2016). Thromboxane A2 receptor (TBXA2R) is a potent survival factor for triple negative breast cancers (TNBCs). *Oncotarget*.

[23] Győrffy B., Surowiak P., Budczies J., Lánczky A. (2013). Online survival analysis software to assess the prognostic value of biomarkers using transcriptomic data in non-small-cell lung cancer. *PLoS ONE*.

[24] Buckley N. E., Haddock P., De Matos Simoes R. (2016). A BRCA1 deficient, NFkappaB driven immune signal predicts good outcome in triple negative breast cancer. *Oncotarget*.

[25] Jézéquel P., Loussouarn D., Guérin-Charbonnel C. (2015). Gene-expression molecular subtyping of triple-negative breast cancer tumours: importance of immune response. *Breast Cancer Research*.

[26] DeNardo D. G., Brennan D. J., Rexhepaj E. (2011). Leukocyte complexity predicts breast cancer survival and functionally regulates response to chemotherapy. *Cancer Discovery*.

[27] Gocheva V., Wang H.-W., Gadea B. B. (2010). IL-4 induces cathepsin protease activity in tumor-associated macrophages to promote cancer growth and invasion. *Genes & Development*.

[28] Vasconcelos I., Hussainzada A., Berger S. (2016). The St. Gallen surrogate classification for breast cancer subtypes successfully predicts tumor presenting features, nodal involvement, recurrence patterns and disease free survival. *The Breast*.

[29] Mulligan J. M., Hill L. A., Deharo S. (2014). Identification and validation of an anthracycline/cyclophosphamide-based chemotherapy response assay in breast cancer. *Journal of the National Cancer Institute*.

[30] Lehmann B. D., Jovanović B., Chen X. (2016). Refinement of triple-negative breast cancer molecular subtypes: implications for neoadjuvant chemotherapy selection. *PLoS ONE*.

[31] Gautam J., Bae Y. K., Kim J.-A. (2017). Up-regulation of cathepsin S expression by HSP90 and 5-HT7 receptor-dependent serotonin signaling correlates with triple negativity of human breast cancer. *Breast Cancer Research and Treatment*.

[32] Crowe J. P., Gordon N. H., Hubay C. A. (1991). Estrogen receptor determination and long term survival of patients with carcinoma of the breast. *Surgery, gynecology & obstetrics*.

[33] Truong P. T., Bernstein V., Wai E., Chua B., Speers C., Olivotto I. A. (2002). Age-related variations in the use of axillary dissection: a survival analysis of 8038 women with T1-ST2 breast cancer. *International Journal of Radiation Oncology, Biology, Physics*.

[34] Carey L. A., Perou C. M., Livasy C. A. (2006). Race, breast cancer subtypes, and survival in the carolina breast cancer study. *The Journal of the American Medical Association*.

[35] Yu K., Wu J., Shen Z., Shao Z. (2012). Hazard of breast cancer-specific mortality among women with estrogen receptor-positive breast cancer after five years from diagnosis: implication for extended endocrine therapy. *The Journal of Clinical Endocrinology & Metabolism*.

[36] Sopik V., Sun P., Narod S. A. (2017). The prognostic effect of estrogen receptor status differs for younger versus older breast cancer patients. *Breast Cancer Research and Treatment*.

[37] Poh A. R., Ernst M. (2018). Targeting macrophages in cancer: from bench to bedside. *Frontiers in Oncology*.

[38] Verdoes M., Edgington L., Scheeren F. A. (2012). A nonpeptidic cathepsin S activity-based probe for noninvasive optical imaging of tumor-associated macrophages. *Chemistry & Biology*.

[39] Shree T., Olson O. C., Elie B. T. (2011). Macrophages and cathepsin proteases blunt chemotherapeutic response in breast cancer. *Genes & Development*.

[40] Yang M., Liu J., Shao J. (2014). Cathepsin S-mediated autophagic flux in tumor-associated macrophages accelerate tumor development by promoting M2 polarization. *Molecular Cancer*.

[41] Salpeter S. J., Pozniak Y., Merquiol E., Ben-Nun Y., Geiger T., Blum G. (2015). A novel cysteine cathepsin inhibitor yields macrophage cell death and mammary tumor regression. *Oncogene*.

[42] Lewis C., McQuaid S., Clark P. (2018). The Northern Ireland biobank: a cancer focused repository of science. *Open Journal of Bioresources*.

