# Supplementary material for: A Novel Role for Cathepsin S as a Potential Biomarker in Triple Negative Breast Cancer
Source: J Oncol. 2019 Jun 27;2019:3980273. doi: 10.1155/2019/3980273 (PMC6620839; doi:10.1155/2019/3980273)
Supplement: Supplementary Materials — Supplementary Figure 1: Flowchart describing study design. Supplementary Figure 2: Breakdown of CTSS scores across subtypes and associated Kaplan-Meier OS figures. (a) Percentage breakdown of patients with high and low CTSS in (i) epithelial and (ii) stromal compartments. Kaplan-Meier curve of (b) Luminal A, (c) Luminal B/HER2-, (d) Luminal B/HER2+, and (e) HER2+ breast cancer subtypes overall survival (OS) stratified based on high or low CTSS expression in (i) stromal and (ii) epithelial compartment. Log-Rank p-value indicated. N=number of patients. Supplementary Figure 3: Complete Lehman analysis figure of subtype CTSS gene expression. Supplementary Figure 4: No effect on OS or RFS in total, Luminal A, or Luminal B breast cancer subtypes stratified by gene expression. Kaplan-Meier curves stratifying (a) total breast cancer patient population, (b) luminal A patient population, and (c) luminal B patient population, based on high or low CTSS gene expression evaluating (i) overall survival (OS) and (ii) relapse free survival (RFS). Log-rank p-value and hazard ratio (HR) indicated. N=number of patients. Supplementary Table 1: Increased CTSS gene expression associated with improved survival in HER2+ and Triple Negative breast cancers. Analysis of publicly available gene expression data with matching clinical outcome from KM plotter revealed a significant association of increased CTSS expression with improved overall survival and relapse free survival. N=number of patients. HR= hazard ratio. CI=95% confidence intervals. [file 3980273.f1.pptx]

## Slide 1
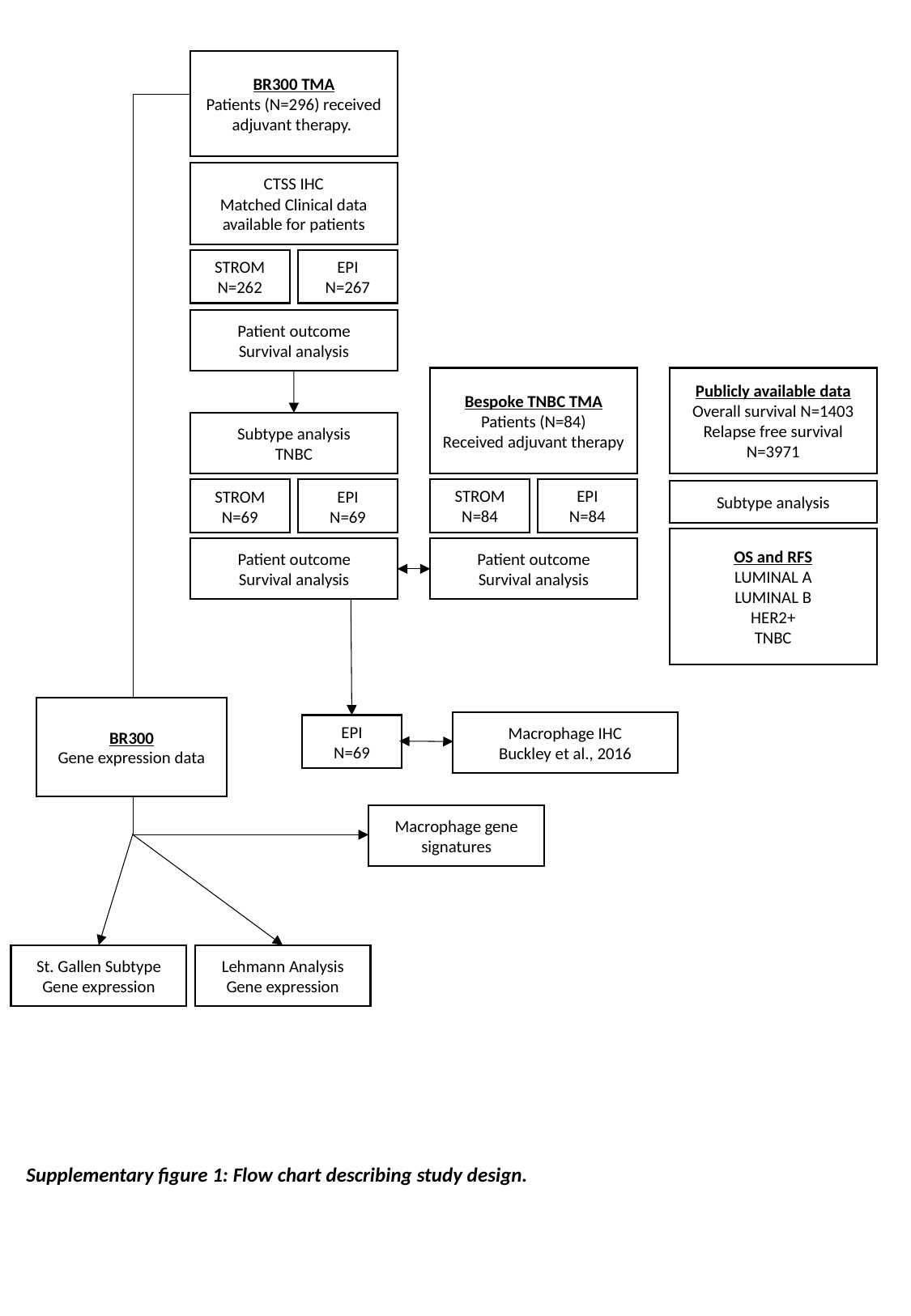

BR300 TMA
Patients (N=296) received adjuvant therapy.
CTSS IHC
Matched Clinical data available for patients
STROM
N=262
EPI
N=267
Patient outcome
Survival analysis
Bespoke TNBC TMA
Patients (N=84)
Received adjuvant therapy
Publicly available data
Overall survival N=1403
Relapse free survival N=3971
Subtype analysis
TNBC
STROM
N=84
EPI
N=84
STROM
N=69
EPI
N=69
Subtype analysis
OS and RFS
LUMINAL A
LUMINAL B
HER2+
TNBC
Patient outcome
Survival analysis
Patient outcome
Survival analysis
BR300
Gene expression data
Macrophage IHC
Buckley et al., 2016
EPI
N=69
Macrophage gene signatures
St. Gallen Subtype
Gene expression
Lehmann Analysis
Gene expression
Supplementary figure 1: Flow chart describing study design.

## Slide 2
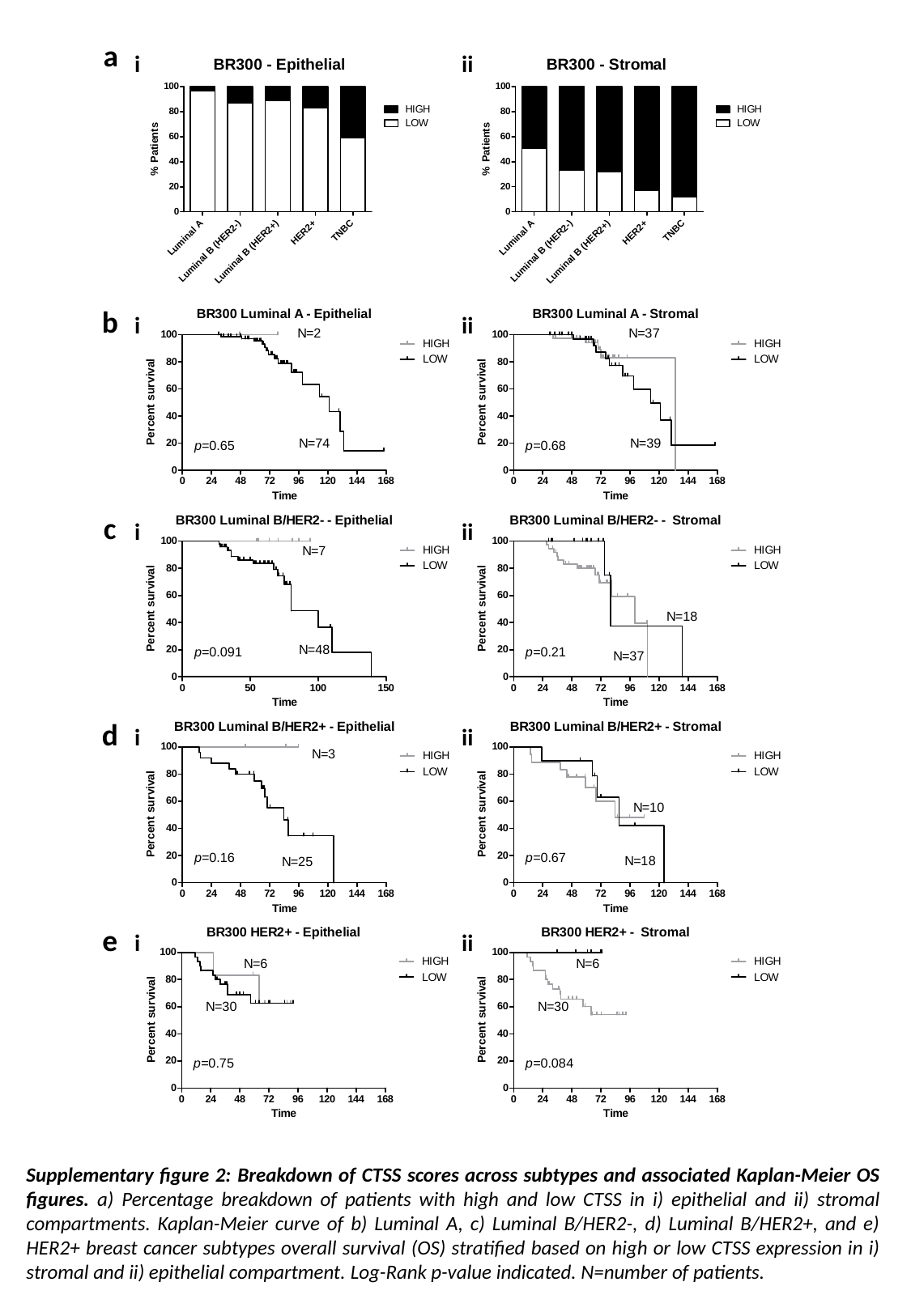

a
i
ii
b
i
ii
c
i
ii
d
i
ii
e
i
ii
Supplementary figure 2: Breakdown of CTSS scores across subtypes and associated Kaplan-Meier OS figures. a) Percentage breakdown of patients with high and low CTSS in i) epithelial and ii) stromal compartments. Kaplan-Meier curve of b) Luminal A, c) Luminal B/HER2-, d) Luminal B/HER2+, and e) HER2+ breast cancer subtypes overall survival (OS) stratified based on high or low CTSS expression in i) stromal and ii) epithelial compartment. Log-Rank p-value indicated. N=number of patients.

## Slide 3
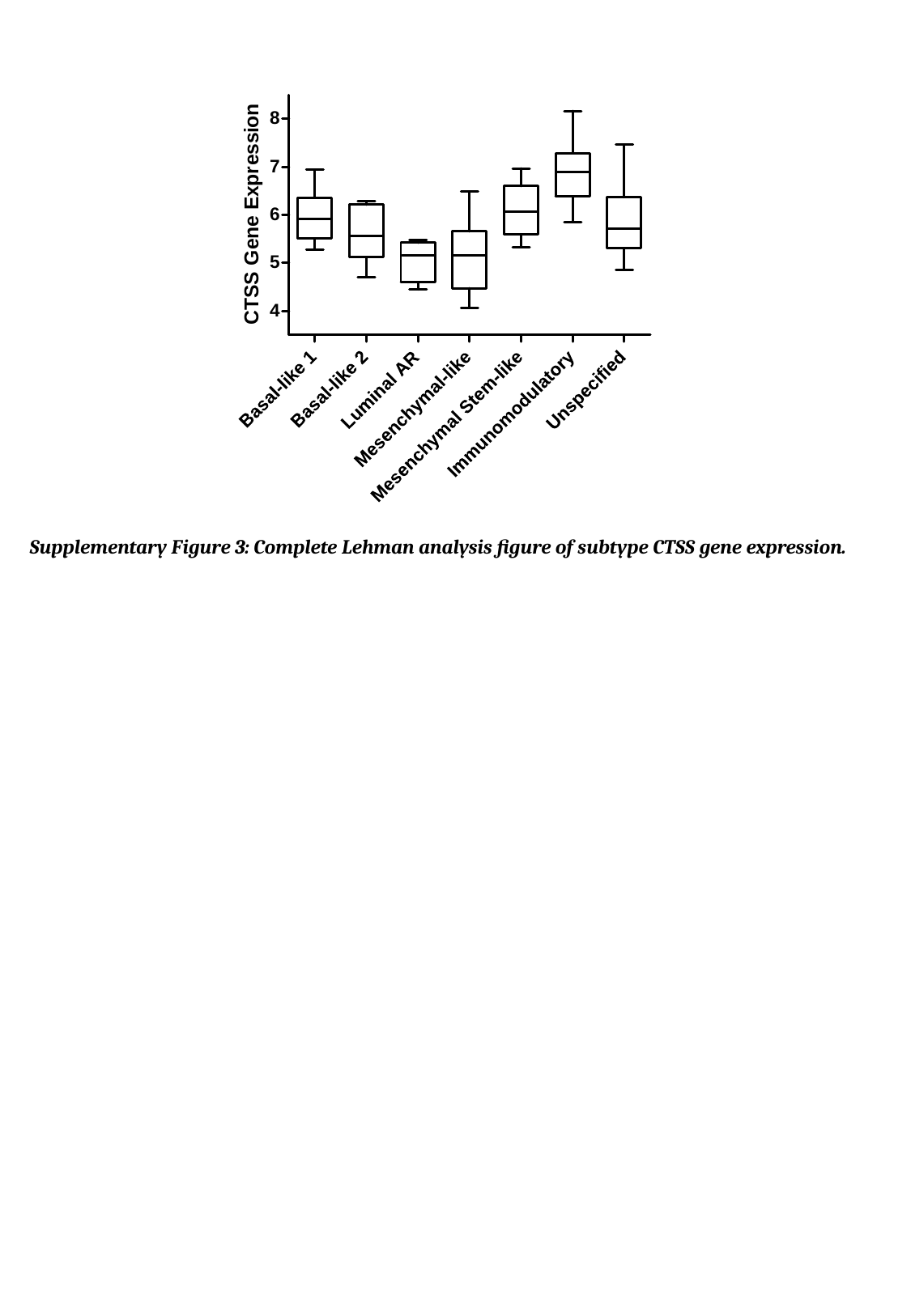

Supplementary Figure 3: Complete Lehman analysis figure of subtype CTSS gene expression.

## Slide 4
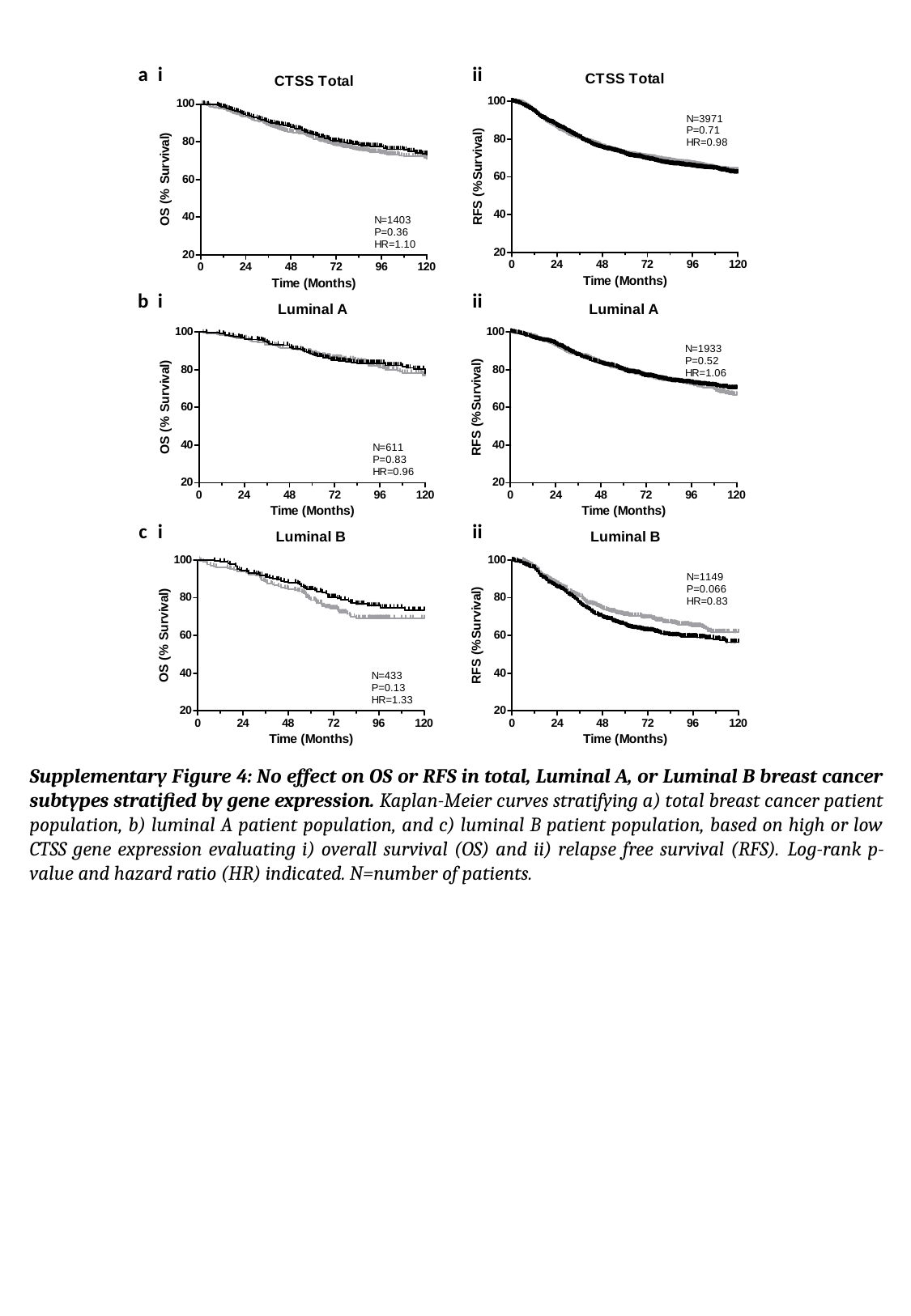

a
i
ii
b
i
ii
c
i
ii
Supplementary Figure 4: No effect on OS or RFS in total, Luminal A, or Luminal B breast cancer subtypes stratified by gene expression. Kaplan-Meier curves stratifying a) total breast cancer patient population, b) luminal A patient population, and c) luminal B patient population, based on high or low CTSS gene expression evaluating i) overall survival (OS) and ii) relapse free survival (RFS). Log-rank p-value and hazard ratio (HR) indicated. N=number of patients.

## Slide 5
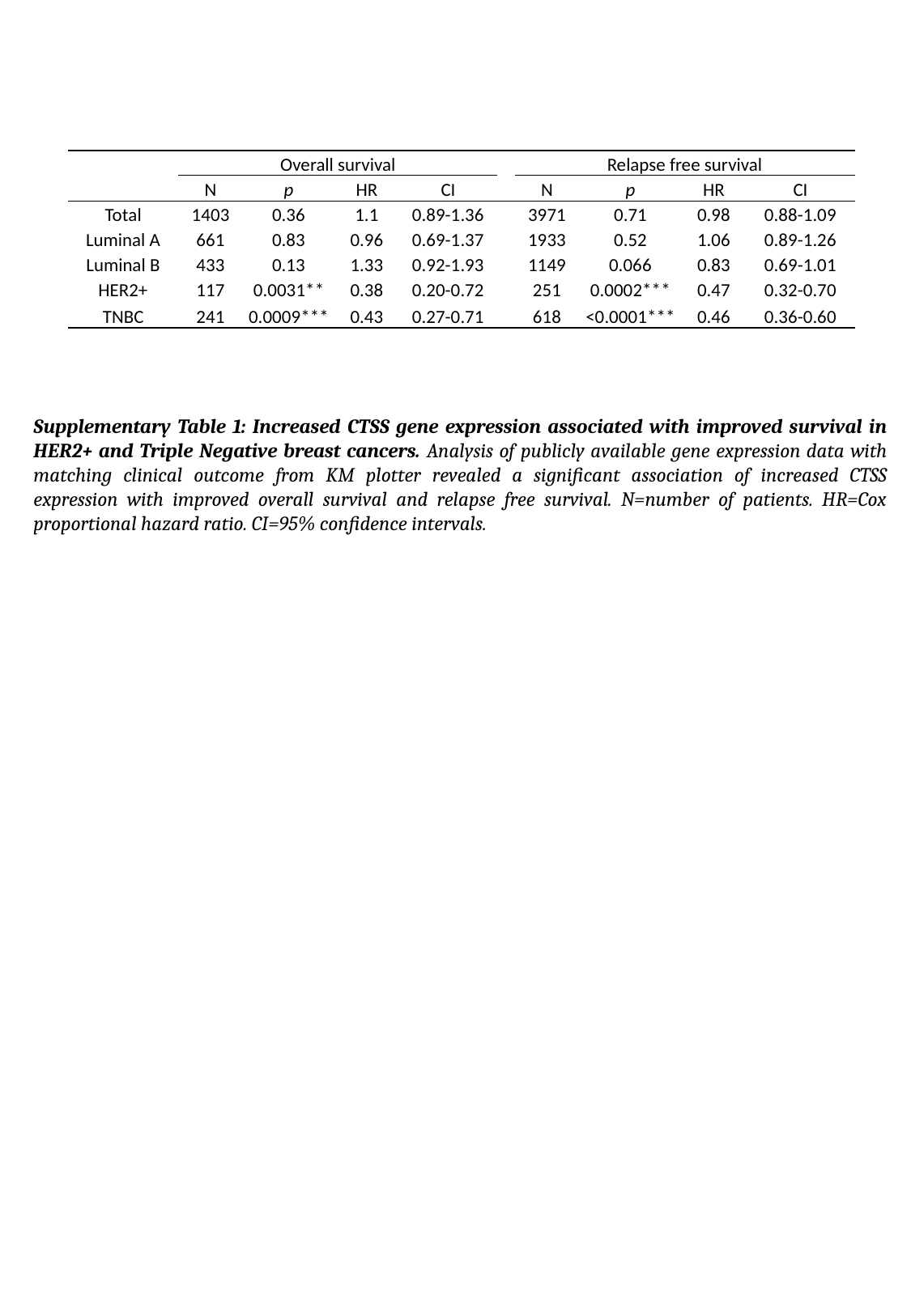

| | | | | | | | | | | |
| --- | --- | --- | --- | --- | --- | --- | --- | --- | --- | --- |
| | Overall survival | | | | | | Relapse free survival | | | |
| | N | p | HR | CI | | | N | p | HR | CI |
| Total | 1403 | 0.36 | 1.1 | 0.89-1.36 | | | 3971 | 0.71 | 0.98 | 0.88-1.09 |
| Luminal A | 661 | 0.83 | 0.96 | 0.69-1.37 | | | 1933 | 0.52 | 1.06 | 0.89-1.26 |
| Luminal B | 433 | 0.13 | 1.33 | 0.92-1.93 | | | 1149 | 0.066 | 0.83 | 0.69-1.01 |
| HER2+ | 117 | 0.0031\*\* | 0.38 | 0.20-0.72 | | | 251 | 0.0002\*\*\* | 0.47 | 0.32-0.70 |
| TNBC | 241 | 0.0009\*\*\* | 0.43 | 0.27-0.71 | | | 618 | <0.0001\*\*\* | 0.46 | 0.36-0.60 |
Supplementary Table 1: Increased CTSS gene expression associated with improved survival in HER2+ and Triple Negative breast cancers. Analysis of publicly available gene expression data with matching clinical outcome from KM plotter revealed a significant association of increased CTSS expression with improved overall survival and relapse free survival. N=number of patients. HR=Cox proportional hazard ratio. CI=95% confidence intervals.
